# Domestic water carrying and its implications for health: a review and mixed methods pilot study in Limpopo Province, South Africa

**DOI:** 10.1186/1476-069X-9-52

**Published:** 2010-08-26

**Authors:** Jo-Anne L Geere, Paul R Hunter, Paul Jagals

**Affiliations:** 1 Faculty of Health, University of East Anglia, Norwich, NR4 7TJ Norfolk. UK; 2 Faculty of Science, Tshwane University of Technology, Pretoria, South Africa

## Abstract

**Background:**

Lack of access to safe water remains a significant risk factor for poor health in developing countries. There has been little research into the health effects of frequently carrying containers of water. The aims of this study were to better understand how domestic water carrying is performed, identify potential health risk factors and gain insight into the possible health effects of the task.

**Methods:**

Mixed methods of data collection from six were used to explore water carrying performed by people in six rural villages of Limpopo Province, South Africa. Data was collected through semi-structured interviews and through observation and measurement. Linear regression modelling were used to identify significant correlations between potential risk factors and rating of perceived exertion (RPE) or self reported pain. Independent t-tests were used to compare the mean values of potential risk factors and RPE between sub-groups reporting pain and those not reporting pain.

**Results:**

Water carrying was mainly performed by women or children carrying containers on their head (mean container weight 19.5 kg) over a mean distance of 337 m. The prevalence of spinal (neck or back) pain was 69% and back pain was 38%. Of participants who carried water by head loading, the distance walked by those who reported spinal pain was significantly less than those who did not (173 m 95%CI 2-343; p = 0.048). For head loaders reporting head or neck pain compared to those who did not, the differences in weight of water carried (4.6 kg 95%CI -9.7-0.5; p = 0.069) and RPE (2.5 95%CI -5.1-0.1; p = 0.051) were borderline statistically significant. For head loaders, RPE was significantly correlated with container weight (r = 0.52; p = 0.011) and incline (r = 0.459; p = 0.018)

**Conclusions:**

Typical water carrying methods impose physical loading with potential to produce musculoskeletal disorders and related disability. This exploratory study is limited by a small sample size and future research should aim to better understand the type and strength of association between water carrying and health, particularly musculoskeletal disorders. However, these preliminary findings suggest that efforts should be directed toward eliminating the need for water carrying, or where it must continue, identifying and reducing risk factors for musculoskeletal disorders and physical injury.

## Background

Improved health-related water management could prevent one tenth of the current global disease burden and investments in improved access to safe drinking water may realize at least ten fold economic returns [1]. Yet lack of access to safe water remains the third most significant risk factor for poor health in developing countries [2]. The health impact of various interventions to improve access to safe water has been extensively reviewed, but primarily by focusing on rates of acute infectious diarrhoeal illness to evaluate outcome [2-5]. It is likely that more health impacts of sub-optimal water supply are frequently overlooked or underestimated, because effects other than acute diarrhoeal illness are not usually considered [1].

Understanding the health impacts of sub-optimal water access more broadly is crucial for appropriate and sustainable water resource development. The benefits of investing in interventions to improve safe water access may be underestimated and, therefore, such interventions not prioritised, if the broader health impacts are not comprehensively evaluated. For example, many people must still collect and physically carry water from a source distant to their home, which may have important health consequences for those who perform the task [6].

Water filled containers are often carried on the head, however, transportation with wheel barrows, animal drawn carts or by rolling filled containers has also been observed [7,8]. These methods obviously create physical demands on the body and the potential for adverse physical stress from regularly carrying loads of water has been recognised [6,9,10]. Physical loading of the body within an individual's capacity for adaptive responses may lead to tissue strengthening, however, frequent loading beyond capacity for adaptation or repair may lead to injury through fatigue failure, accumulation of fatigue damage [11] or early degenerative changes in bone and soft tissues [12].

Assumptions have been made that water carrying is detrimental to health and associated with musculoskeletal disorders, such as spinal pain or other joint problems [8,13,14]. Such assumptions are supported by strong evidence that the physical demands of work such as handling heavy materials, bending, twisting and lifting, are risk factors for onset of simple low back pain [11,15] and other musculoskeletal disorders [16,17]. In particular carrying heavy loads on the head by professional porters has been documented to cause catastrophic injury, such as spinal fracture, dislocation or death [18] and has also been associated with early onset of degenerative changes in the cervical spine [12,17,19,20].

Although head loading due to occupational activities has been associated with degenerative changes in the cervical spine, the relationship between symptoms such as neck pain and activities which require head loading is not clear. Despite a much higher prevalence of upper cervical osteoarthritis in porters (91.6% in male porters compared to 6.8% in the control group) Badve et al. [17] stated that an association between symptoms and radiological changes was not found. Similarly, a recent systematic review did not find evidence that cervical disc degeneration is a risk factor for neck pain and reported variable evidence for a relationship between radiographic signs of degeneration and neck pain prevalence [21]. A recent study found that degenerative changes observed in cervical plain films were poorly related to the severity of symptoms or neck dysfunction in women with chronic pain and working in sedentary occupations [22].

However, very few studies have specifically investigated water carrying as it is performed by women and children in developing countries and used appropriate methodologies to investigate its association with health generally or musculoskeletal disorders specifically [10,23,24]. Most studies investigating the health impact of physical loading are of male adult workers [17,25-27] or are situated in high income countries [28] such that existing evidence may not be applicable to women and children who typically collect and carry water for domestic use [29]. Importantly, women and children have reduced injury tolerance for physical loading through the cervical spine compared to men [30-36] and in rural areas may be particularly vulnerable to physical injury due to high levels of poverty, poor health and chronic disease [37-42]. Therefore, it is not clear whether regularly carrying containers of water for domestic use leads to detrimental effects such as accelerated degenerative changes in the spine and other joints and whether or not any such effects are symptomatic and impact on health related quality of life.

Two recent reports indicate that some people may experience high rates of perceived exertion and pain sufficient to limit their capacity to carry water containers [7,43]. Reduced capacity of women or children to collect water due to pain or fatigue may have serious implications for the health of their families. However, water carrying is a physical activity which might also lead to beneficial health effects in some individuals. In researching the health impacts of water carrying, it is important to consider health impacts broadly [7] and recognise the limitations of applying existing evidence to this special activity and population group. Researcher assumptions about risk factors and health effects may introduce bias into research methodology in terms of determining the questions asked and outcomes measured and consequently how study participants report the health impacts of water carrying. For example, the use of leading questions or outcome measures which assume an association with symptoms such as pain might influence participant responses and their description of the health effects of water carrying.

As there is a lack of empirical data specifically related to water carrying, the aims of this study were to better understand how water carrying is performed and experienced by people who perform the task, identify health risk factors potentially related to carrying water and gain insight into the possible health effects of the task. The following research questions are addressed in this report

• Who carries domestic water sourced outside of the home?

• How do people carry domestic water?

• What factors considered to a pose risk of injury or disease in higher income countries and occupational settings, are people exposed to during water carrying?

• Are reports of pain during qualitative interview and ratings of perceived exertion during water carrying correlated with exposure to water carrying related risk factors?

• Are there significant differences in exposure to potential water carrying related risk factors between people who report pain during qualitative interview and those who do not?

• How does pain impact on the ability to carry water?

## Methods

A mixed methods approach was taken, utilising both quantitative and qualitative data to better understand domestic water carrying as it is performed and experienced by adults and children in Limpopo Province, South Africa. Ergonomic principles were used to develop the approach to quantitative data collection. An 'ergonomic' evaluation of work incorporates assessment of a broad range of potential risk factors related to the environment, organisation of work, the nature of the task or the individual [44].

Qualitative enquiry in this study was influenced by the principles of phenomenology as described by Creswell [45] and used to explore the lived experience of water carrying. Individuals with direct experience of water carrying will have unique understanding of the task and can provide insight into how it might impact upon their own health and functioning. As the health effects of water carrying are unknown and might be experienced and interpreted variably by different individuals, such insights can indicate the domains of health which are relevant to people who perform water carrying and, therefore, important to evaluate for a potential association with the activity.

This report will focus on the analysis of the quantitative data, combined with some specific findings from content analysis of the qualitative data generated during individual semi-structured interviews. This approach was used to evaluate the relationship between pain, which was a specific health outcome revealed to be of concern to many of the study participants, and potential risk factors observed to occur during water carrying. More extensive and detailed analysis of the qualitative data will be reported separately [7] and will incorporate the findings of additional data generated from 'natural informal group interviews' which were conducted according to the methods described by Green and Thorogood [46].

### Sampling strategy, participant recruitment and consent

Data was collected from six villages in Limpopo Province, South Africa. Limpopo was chosen as the study area because it is a district with high levels of poverty and where suboptimal water supply is likely to have considerable health impact [47]. It is also a region which is broadly comparable with other poor rural districts of South Africa and other developing countries. The predominant cultural group in the area are the Venda people.

The six villages in the study area were visited on two occasions; over a three-week period in March 2008 and a two-week period in October 2008. The first period was for initial qualitative and quantitative data collection. The second period was to feedback preliminary study findings to participating communities, create an opportunity for community members to comment on the initial interpretation of qualitative data and explore levels of support for future research into water carrying.

The villages were purposively selected to include a range of water service situations and environments which might have different physical effects or expose people to different risk factors for injury or disease. Villages and the water source points within them were chosen to include variations in terrain which might influence methods and effects of water carrying in different ways. For example, many people in one village relied on water sourced from a mountain spring, accessible via steep, slippery and rocky footpaths. Another village, located on a flat plain relied mainly on communal taps accessed via sandy pathways or roads.

Before commencing research, permission for the researchers to work in each village was sought from the 'headman' of each village by the research assistant (RA), a twenty-nine year old Venda male, fluent in several languages including Venda and English and intimately familiar with local customs. All headmen gave verbal permission for the researchers to access their village. Each village was then visited over a period of two to three consecutive days by the principal investigator (JG) and the RA, during which qualitative interview data and quantitative observational data was gathered. Work was ceased in each village when qualitative and quantitative data had been collected from a sample with representation of people with a range of ages, of each gender and with variation in the terrain, type of path and distance over which they walked to collect water. In each village, specific water source points were chosen according to what was available in the village and to include representation in the study of varying water sources (a river, natural springs and communal taps) and infrastructure (e.g. water pumping station overflow pipes or communal taps with differing construction design).

People observed to be intending to collect water were initially approached by the RA and briefly informed in their preferred language of the study purpose and procedures. Those willing to participate were provided with more detailed explanation of the study both verbally and with participant information sheets written in TshiVenda. It was assumed that all participants may have had poor literacy skills as it was not possible to evaluate the literacy level of each participant in the field. Therefore the study purpose and procedures and the request for voluntary participation were fully explained verbally to all participants in their preferred language. Participants were also provided with information and consent forms written in their preferred language and an 'easy to read' version which included graphic illustrations rather than any sections of lengthy text. Both versions of the information and consent forms were translated from English into Venda by the RA and then independently back translated by a local native Venda speaker. The back translation indicated that conceptually accurate and meaningful translation of the documents was achieved.

If informed voluntary consent was granted, consent forms were signed and individuals were recruited to the study. Where children were observed to collect water with an adult relative or guardian, informed signed consent for the child to participate was sought from the adult. Agreement was also sought verbally from the child in a non-coercive manner by the RA, who as a Venda male was sensitive to culturally appropriate ways to interact with the children. Care was taken by the principal investigator and RA to monitor from children's behaviour that they were not adversely affected by participating in the study. No behaviour to indicate that any adverse effects occurred as a result of participation in the study was observed.

Although five children collected water in the company of an adult, eleven collected water without adult supervision. In such instances, the study purpose and procedures were first explained to the children by the RA in a manner appropriate to their age and level of understanding. Once voluntary verbal agreement was obtained from the children, measurements of their weight and height and the weight of filled containers they intended to carry were taken. They were then video recorded and observed while filling containers and carrying water from the collection point to their home. On arrival at the house, a parent or adult guardian was identified through discussion conducted in Venda between the RA, child and adults present. The adult identified in this way as guardian for the child was advised of the study purpose and procedures, and formal written consent for the child's participation sought. This created opportunity for the video capture and observational data to be erased in the event of the parent or guardian not consenting to participation of their child, however, such a situation did not arise.

Of those invited to participate in the study only three declined. Forty-three people were recruited to the study for collection of observational data and/or semi-structured interviews. Four participated in semi-structured interviews (one female child, two women and one man) but were not observed carrying water, leaving a total sample of 39 people from whom observational data was collected (Table 1). Twenty-nine of the people observed carrying water were also participants in semi-structured interviews, purposively chosen to meet the inclusion criteria and ensure representation of males and females with a range of ages from each village (Table 2). Ethical approval for the study was obtained from the International Development Ethics Committee, University of East Anglia, Norwich and the Higher Degrees and Ethics Committee for the Faculty of Health Sciences, University of Johannesburg.

**Table 1:** Participant demographics all water carrying methods (n: 39)

	Mean (sd)	Minimum	Maximum
Age (years)	25 (15.5)	6	64

Height (cm)	151.49 (17.55)	110	176

Weight (kg)	49.55 (21.74)	16	106

BMI	20.53 (6.32)	13.15	41.41

Female: male	34:5		

Adults (F, M): children (F, M)	(22, 1): (12,4)		

A children: U children	5:11		

F: female; M: male; A: children accompanied by an adult during water carrying; U: children unaccompanied by and adult during water carrying

**Table 2:** Participant numbers per village and data collection methods

Village (population)	Water system	Alternative water sources	Observed carrying water	**Observed carrying water & SSI**^**1**^	**Observed carrying water & NGM**^**2**^
1 (2,830)	28 CT^3^	River, mobile water tanker	9	8	1

2 (2,457)	43 CT	Stream or borehole	5	5	0

3 (5,286)	45 CT	River, canal, borehole or pumping station over-flow pipe	13	8	3

4 (1,129)	2 springs	Plastic water tank filled by water tankers	8	6	2

5 & 6 (719)	23 CT	River, spring or borehole	4	1	3

^1^SSI = semi-structured interview; ^2^NGM = Informal natural group meeting; ^3^Communal Taps

### Inclusion and exclusion criteria

To be included in the study, people were:

• Male or female adults or children of any age

• Individuals usually residing within the study villages and providing informed voluntary consent to participate

• Individuals physically carrying or intending to physically carry water containers as part of their usual activities

People were excluded from participation if they:

• had no personal experience of carrying water for domestic use

• were using methods of transporting containers which did not involve them physically carrying the filled water containers from the water source to a home, for example through use of donkey carts or motor vehicles

### Data Collection

Demographic data and information on the usual frequency and quantity of water carried was obtained from each participant or their guardian verbally and documented in a recruitment form and structured observation form. Qualitative data reported in this paper was collected through semi-structured interviews according to the methods described by Green and Thorogood [46]. Participant's verbal accounts, or 'self-report' [48] of their own experiences of water carrying were fully audio-recorded during semi-structured interviews which were conducted in a location chosen by the participants near to or in their own home. The interviews were conducted using open interview guide questions such as 'Can you tell me about your experiences of carrying water?' or 'How do you think carrying water affects you?' to reduce researcher influences on the type of health impacts discussed by participants. The interview discussions were conducted with immediate verbal translation between Venda and English (on one occasion between Pedi and English) performed by the RA, to facilitate communication between the RA, principal investigator and participant. The English questions and the RA's English translation of the participants' responses were fully transcribed.

Quantitative data were gathered from each participant's verbal report in response to a set of structured interview questions, as well as simple measurements and observation. A tape measure was used to measure each participant's height, using a level flat standing-platform and a clipboard placed horizontally on the head to provide level points for measurement. The weight of participants as well as that of the filled water containers they carried was measured in kilograms using bathroom scales and calculated from the mean value of three consecutive weighing scores to reduce measurement error. The principal investigator and RA observed the manner in which participants carried water from the source point to their home. Observations were recorded through video capture, photography and documentation in field notes. Specifically, time taken for the water carrying trip from source to home, body postures adopted during lifting and handling as well as while carrying containers, carrying methods and the environment in which water carrying occurred were captured with video-recording using a Panasonic Mini-DV digital video camera (Model NV-GS320). A GPS unit (Garmin CSX 60) was used to measure the distance (in metres) travelled from the water source to the home in one direction whilst carrying a filled water container.

The modified Borg scale (RPE) [49] was used to gain insight into the intensity of work performed by study participants. The modified Borg scale is a twelve grade category rating scale with ratio properties, which combines verbal and numerical descriptors that can be used to measure a person's rating of their perceived exertion during a specific task [49]. A numeric score of 0 equates to a verbal descriptor of 'nothing at all', 10 to 'very, very strong' and 12 to 'maximal'. It has been validated for used in diverse populations and used with Xhosa speaking women carrying containers of water in a laboratory setting [43]. In this study participants were presented with a printed Venda version of the scale which was verbally explained to them by the RA. They were asked to estimate the sensation of the effort required for carrying water immediately on completion of a water carrying trip and to point to or choose the verbal descriptor or number most closely matching their sensation of effort.

Qualitative and quantitative data collection procedures were piloted in the study area with a Venda speaking woman during and immediately after a water carrying trip. This was done to ensure that interview questions were easily understood and facilitated relevant discussion and that measurement methods to collect quantitative data were feasible for use in the field.

### Data Analyses

Quantitative data were entered into SPSS 15.0 and descriptive statistics were generated for all participants observed collecting water and for participants carrying water by head loading (Table 3).

**Table 3:** Descriptive statistics for all water carrying methods and for head loaders only

	All water carrying methods				Head loaders only			
	**No**.	**Mean (sd)**	**Min**	**Max**	**No**.	**Mean (sd)**	**Min**	**Max**

Distance (m)1	35	330 (178)	40	650	29	337 (190)	40	650

Total weight carried (kg)	33	28.9 (22.8)	4	111				

Container weight (Newtons)					27	191 (60)	39	265

Number of containers carried	39	1.4 (1)	1	5	30	1	1	1

Filled container weight (kg)	33	20.2 (6.7)	4	27.8	27	19.5 (6.1)	4	27

Container Weight/Body Weight (%)	33	58.7 (42.7)	16.3	200.7	27	41.4 (14.6)	16.3	77.8

Carry time per trip (minutes)	37	6 (4)	1	15	29	6 (4)	1	15

TDCT2 minutes	22	18 (13)	1	46	17	18 (13)	1	45

Frequency water collection per/day	24	3.4 (2)	1	8	18	3.4 (2.2)	1	8

Frequency water collection days/week	24	6 (2)	1	7	20	6 (2)	2	7

Rating of Perceived Exertion (RPE)	35	7 (3)	2	10	26	7 (3)	2	10

^1^Distance is reported in meters as that from the water source to the home in one direction whilst carrying a filled water container; ^2^TDCT total daily carrying time = observed carrying time multiplied by reported usual daily frequency of water carrying

Content analysis of participant responses recorded in 29 semi-structured interview transcripts was used to identify participants complaining of pain in particular body regions and used to calculate the prevalence of spinal pain (defined in this study as self reported head, neck or back pain), back pain and neck pain. It was also used to determine subgroups of participants who did and did not report pain, for comparative statistical analysis of other variables.

Two techniques were used to gain insight into the level or intensity of work which the participants performed. Firstly, the participants rated their level of perceived exertion using the modified Borg scale. Secondly, the weight of water carried (kg) was calculated as a percentage of body weight for all carrying methods and in Newtons of force (N) for head loading. Force in Newtons (N) is equal to mass (kg) multiplied by gravity (9.8 m/s^2^) such that 1 kg is approximately equal to 9.81 N [11]. The force generated by an object of a known weight carried on the head can therefore be calculated using a simple biomechanical model as described by Oatis [50], if the container is assumed to be in static equilibrium. The forces generated during head loading are simplified in this study and assumed to be the force generated purely by the weight of the water and container carried, directed vertically downward onto the head and spine, with no moment arm.

For analyses of the videoed material, the task of collecting and carrying water in containers was divided into four subtasks: 1) preparing and filling, 2) lifting, 3) carrying and 4) lowering and placement of containers. The video material was analysed by a musculoskeletal physiotherapist (JG) with 21 years of experience in the clinical assessment of human movement and musculoskeletal function, including task and postural analysis. The analysis was performed to distinguish between sub-tasks and for simple visual observation of the whole body postures and movements commonly occurring during water carrying. Specific criteria developed in this study (shown in Additional file 1) to visually identify and record cut-off time points between the subtasks were applied on two separate occasions and the time taken for each subtask calculated twice to minimise simple calculation errors. The two calculated times for each subtask, were then used to generate an average subtask time value for each participant.

Linear regression modelling was used to identify significant correlations between variables and RPE or self reported pain. Information on self-reported pain was drawn from content analysis of transcripts generated from audio recordings of qualitative semi-structured interviews held with 29 participants.

Sub-group analysis was performed on the 21 of 29 interviewed participants who performed water carrying by head loading by grouping those who reported spinal pain and those who did not, as well as participants who reported head/neck pain and those who did not. There were insufficient numbers for sub-group analysis of study participants using other methods of carrying water. Independent t-tests were used to compare the mean values of container weight, distance, carrying time, total daily carrying time (observed carrying time × reported usual daily frequency of water carrying), container weight as a percentage of body weight (CW/BW%) and RPE between the groups.

## Results

### Methods of carrying water

Three methods of carrying water were observed. These were 1) head loading of water-filled containers (n = 30), 2) rolling a water-filled drum (n = 2) and 3) pushing a wheelbarrow weighted with filled water containers (n = 7). Women most commonly used head loading to carry water, 28 of 34 (82%) females compared to two of five (40%) of males. The two boys observed head loading were walking along steep and rocky pathways.

### Potential health impacts of domestic water carrying

Pain was commonly reported as an effect of carrying water in semi-structured interviews. Of the 29 participants, 20 (69%) reported spinal pain, defined in this study as pain reported or indicated through gesture by participants to be in the head, neck, thoracic or lumbo-sacral region during qualitative interview. Of these 11 (38%) reported back pain and 12 (41%) neck or head pain.

### Potential health risk factors

#### Individual factors

The age range of participants was six to 64 (Table 1). Only women and children (aged 16 or less) were observed carrying water, other than one 18 year old unmarried man. Initial analysis of qualitative data supports that water carrying is usually a woman's task, performed by men only when there are no women or female children available to collect water for them.

'male wont collect water, female has to collect water, but its not everybody who support the ideas. There are also possibility in other household that you find the male people without girls so those males has to go an collect water' (young girl, informal natural group meeting 8)

#### Weight of water carried

**Figure 1:**
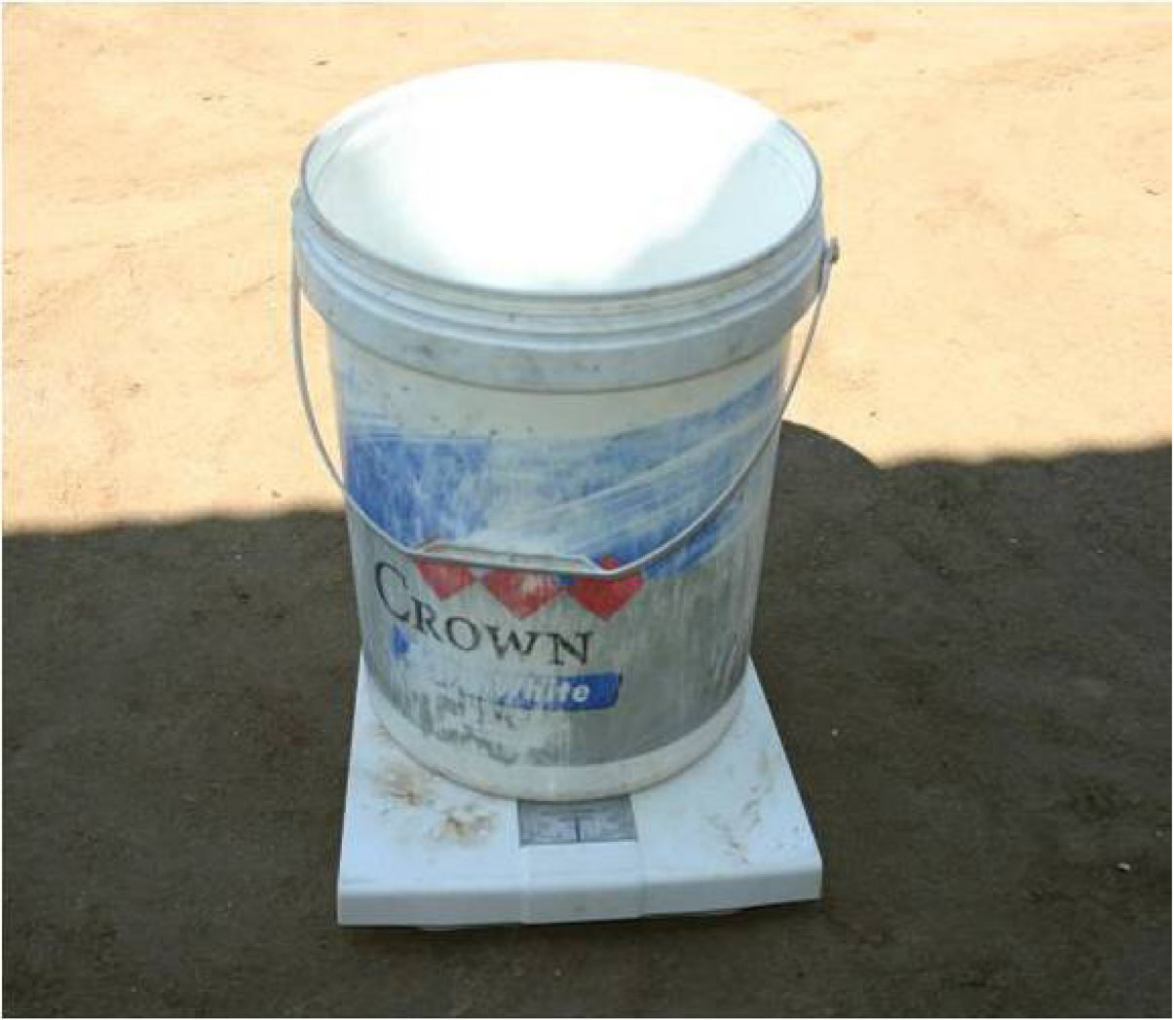
**Typical container used for carrying water**.

The most commonly used containers were fully filled 20 to 25 litre plastic buckets or drums (Figure 1). Because of the head loading method, women typically carried one container per trip. However if using a wheelbarrow, people carried up to five containers, so that although the mean individual container weight for all water carrying methods was 20 kg with a maximum of 28 kg, the mean total weight carried was 29 kg, ranging up to 111 kg (Table 2). For all carrying methods, the mean filled container weight as a percentage of body weight was 59%, with a maximum weight transported by wheelbarrow at 200% of body weight (Table 2). For head loading the mean container weight as percentage of body weight value was 41% ranging from 16 to 78% (Table 3).

**Figure 2:**
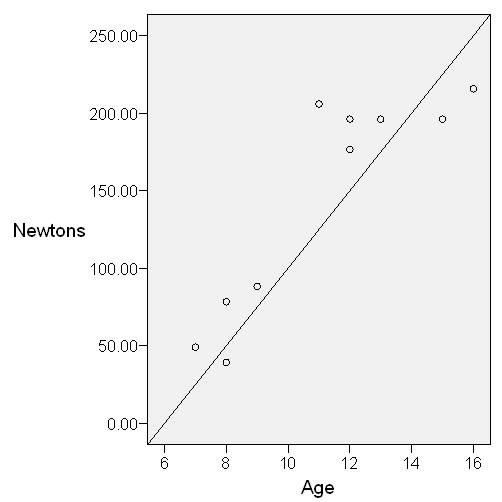
**Scatter plot showing a strong correlation between force due to weight of water against age of children head-loading containers**.

The mean container weight carried by head loading was 19.5 kg (maximum 27 kg), indicating that due to the weight of water alone, this method generated a mean of 191 Newtons (N) and up to 265N of compressive force through the cervical spine (Table 3). Of the children observed carrying water, older children tended to carry higher container weights and therefore higher loading forces (Figure 2).

#### Equipment and environmental factors

The containers and carrying equipment were generally in poor condition and not suited to the environment, for example, wheelbarrows to suit adult physical proportions and with completely worn and damaged tyres were used by very young children on sandy pathways. Container sides were smooth and often wet, making them difficult to grasp securely, particularly as they usually had inadequate or absent handles.

The environment presented potential safety hazards and many physical obstacles to lifting and carrying filled water containers. Most participants, including young children, completed part of their journey on a road way. Particularly at non-tap water sources, such as a river or springs, footpaths were narrow and slippery and required walking across uneven sandy and/or rocky ground. For example, sections of one 'footpath' were actually a stream bed coursing down a steep hillside from a natural spring.

I: 'can you tell me about your experiences of carrying water?'

T: 'The bad thing might be accidents that happens when you have carried the water and you just hit the road and the stone on the road they have possibility that you might fall with the container on your head, that's something that is very bad by carrying water.' ((T: translated response, participant 2, 39 year old woman; I: interviewer question)

'what I can say is that the containers are heavy to me, when it is raining we slip on the way when we come back, we've gotten a problem of the knees when we walk down the hill that its painful, the necks also are painful too, even though you have (gotten) a container on top of the head, the shoulders become painful because they have to lean on that container and it become painful too' (translated response, participant 37, 55 year old woman)

**Figure 3:**
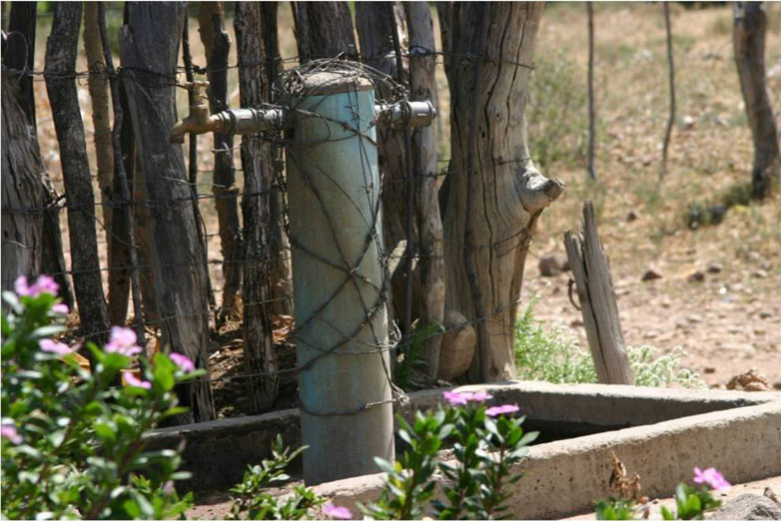
**Communal tap and concrete surround**.

Physical obstacles included barbed wire fences, raised and often worn, jagged edges of concrete platforms at taps (Figure 3), gates, large rocks and pipes as well as other containers, people, equipment and vehicles. Communal taps were most often positioned at a low height and usually required awkward body posture, such as full spinal bending, to lift a filled container up onto the head from ground level. Use of awkward posture was also evident when containers were stored at ground level, or placed inside dwellings with low doorways.

#### Distance

The mean distance over which water was carried by all water carrying methods was

330 meters and ranged from 40 to 650 meters (Table 3). Of interviewed participants who carried water by head loading, the distance walked by those who reported spinal pain was significantly less than those who did not (173 m 95%CI 2-343; p = 0.048; equal variances not assumed) (Table 4). This might represent pain related disability. Preliminary analysis of qualitative data supports that pain may be related to functional disability which may impact on other family members including the ability to carry water, as illustrated in quotes from adult Venda women who participated in semi-structured interviews.

**Table 4:** Subgroup analysis: head loaders with/without report of spinal pain.

	Spinal pain	N	Mean (sd)	Std. error	Mean difference (95% CI)	P value
Container weight (kg)	No	4	13.7 (8.5)	4.2	8.2 (-21.4-4.9)	0.146

	Yes	15	22.0 (3.2)	.8		

Distance (m) ^1^	No	5	470 (132)	59	173 (2-343)	0.048

	Yes	15	297 (188)	49		

Carrying time (min)	No	5	7 (1)	37.2	2.6 m (10sec-5 m)	0.038

	Yes	15	5 (4)	58.7		

TDCT (min) ^2^	No	4	22 (7)	3.5	7.1 (-5.5-19.8)	0.240

	Yes	9	15 (14)	4.5		

CW/BW%	No	4	40 (19)	9.3	2 (-29.6-25.7)	0.856

	Yes	15	42 (15)	3.9		

RPE	No	4	6.2 (4.3)	2.2	1.0 (-7.6-5.6)	0.681

	Yes	15	7.3 (2.6)	0.2		

^1^Distance is reported in meters as that from the water source to the home in one direction whilst carrying a filled water container; ^2^TDCT total daily carrying time: equals observed carrying time multiplied by reported usual daily frequency of water carrying; CW/BW%: container weight as a percentage of body weight; RPE: rating of perceived exertion measured with the Modified Borg Scale

'sometimes it happens that after collecting all eight containers, and filling that big drum its become a problem for her that she couldn't even cook or she couldn't do another work so, she will have to wait for the kids to cook for her and bring some water for her because of what happened just after she collected water' (translated response, participant 10, 33 year old woman)

T: 'the container pressurise my neck as my neck has to hold the head and that container on top, and then by so doing that when the container pressurise me it affect my neck in such a way I feel pain when I just arrive at home... I think it takes a lot of my time because I was supposed to look after these babies making food for them, taking care for the family and also myself rather than to go and collect water, but due to the fact of I have to collect water it takes a lot of my time'

(T: translated response from participant 39, 31 year old mother of five children including four month old triplets; I: interviewer question)

'yes it does affect me sometimes because when I went to some farming and helping on planting some tomato and chillies I have to come back late afternoon and go and help and collect some water, then my body's painful. I cannot collect more water such as I want to collect so that's another problem that collecting water it's affecting me' (translated response, participant 20, 38 year old woman)

#### Rating of Perceived Exertion

The RPE score ranged from two to ten with a mean value of seven for water carrying by head loading (Table 3), as well as when all methods were included in the analysis (Table 2). For head loaders, RPE was significantly correlated with container weight (r = 0.520; p = 0.011) and incline (r = 0.459; p = 0.018). This suggests that the volume of water carried and environmental factors, particularly the incline or gradient of the path along which water is carried, are likely to influence the physical work of water carrying as indicated by RPE. For head loaders reporting head or neck pain, the differences in weight of water carried (4.6 kg 95%CI -9.7-0.5; p = 0.069; equal variances not assumed) and RPE (2.5 95%CI -5.1-0.1; p = 0.051; equal variances not assumed) were borderline significant (Table 5).

**Table 5:** Subgroup analysis: head loaders with/without report of head or neck pain.

	Head or neck pain	N	Mean (sd)	Std. error	Mean difference (95% CI)	P value (2 tailed)
Container weight (kg)	No	10	18.06 (6.5)	2.05	4.61 (-9.72-0.49)	0.069

	Yes	9	22.68 (3.4)	1.14		

Distance (m)	No	12	334 (209)	60.3	15.8 (-19.4-16.2)	0.854

	Yes	8	350 (167)	59.2		

Carrying time (min)	No	11	6 (3)	0.9	12.7 sec (-3.4-3.8m)	0.901

	Yes	9	5 (4)	1.4		

TDCT (min)	No	7	17 (12)	4.4	0.16 (-16.1-15.8)	0.983

	Yes	6	17 (14)	5.6		

CW/BW%	No	10	39 (15)	4.8	5.6 (-20.7-9.6)	0.448

	yes	9	45 (16)	5.3		

RPE	No	11	6.0 (3)	0.9	2.5 (-5.1-0.1)	0.051

	Yes	8	8.5 (2)	0.8		

^1^Distance is reported in meters as that from the water source to the home in one direction whilst carrying a filled water container; ^2^TDCT total daily carrying time: equals observed carrying time multiplied by reported usual daily frequency of water carrying; CW/BW%: container weight as a percentage of body weight; RPE: rating of perceived exertion measured with the Modified Borg Scale

## Discussion

The prevalence of back pain among this mixed group of children and adults, at 38% was higher than that reported in two South African studies included in a recent review [27], and which reported point prevalence for low back pain of 14% for children and 25% for adults. Importantly, we may have underestimated the prevalence of pain in the study sample due to our data collection methods. In keeping with a phenomenological approach, open questions about the health effects of water carrying were asked during semi-structured interviews to capture the potentially varied impacts which people who carry water might perceive the task to have. Participants complaining of pain were identified from their responses to the open interview questions and therefore volunteered pain as a health effect without direct prompting or suggestion that it would be linked to water carrying. In most studies investigating pain, structured outcome measures which directly ask about pain intensity or quality are used. Such direct questions may encourage pain reporting which might not be recalled or mentioned in response to more open interview questions.

A recent Danish study found that women are more likely to report spinal pain than men [51], therefore it is possible that the high proportion of women in this study, due to their role as water carriers, may explain the high prevalence of self reported pain. However, reasons for a potential association between pain reporting and gender may be different in this study population and are as yet unknown. It may be relevant that women in sub-Saharan Africa are disproportionately affected by HIV disease. HIV is associated with rheumatological conditions such as reactive arthritis [37], osteoporosis, fragility fractures and impaired fracture healing [42] and a high prevalence of pain, linked with significant psychological and functional morbidity [39]. How and why pain is reported will vary in different cultural and social contexts [15] and the relationships between physical, psychological and social influences on pain reporting amongst Venda women have not been determined. Future research should investigate the association between bio-psychosocial factors, co-morbidity and pain reporting amongst women who carry heavy water loads as well as pain impact, through participant ratings of pain intensity, duration, frequency and pain-related disability.

This study supports Cleaver's [52] claim that males more commonly use methods of water carrying which utilise equipment. However, in this study, two boys who used a steep and rocky pathway, which made use of any transportation equipment such as a wheelbarrow impossible, were also observed to carry water containers on their heads. Therefore, environmental factors such as path quality and incline gradient may also determine which carrying methods are used. Generally, this study suggests that women and children carry water and women are more likely to carry water in a way (head loading) which will focus and transmit forces through the cervical spine.

Others have reported load-weight as a percentage of body weight and tested for its association with outcomes such as self reported pain [53]. In the United States Moore et al. [53] concluded that backpack weights for children should remain below 10% of body weight and a recent review reported recommendations from several authors that back pack weights for children should be limited to 10-15% of body weight or less [54]. However conclusions drawn from studies set in high income countries may not be generalisable to poorer rural communities, where factors such as childhood health, development and general levels and types of physical activity are likely to differ in significant ways. Nevertheless, in comparison, the high container weights in proportion to body weight carried by women and children in this study seem a potential risk factor for self reported pain. A recent South African study found that a large majority of children who collected water and reported that their health had worsened complained of neck or back pain [23].

Compression forces generated purely by the weight of water carried through head loading in this study may be unlikely to exceed tissue tolerances described in cadaveric studies [30,36,55,56], if applied briefly during a single loading occasion. Older children tended to carry heavier loads than the younger children in the study and their tolerance limits may be closer to those of adults. However, injury tolerance limits based on cadaver studies can only provide estimates of living tissue strength [31] which may be reduced by factors such as malnutrition or chronic illness [57], both of which are highly prevalent in poor rural areas [58] such as can be found in Limpopo Province. In particular, individuals living with HIV disease may suffer from osteopenia and are known to be more at risk of fragility fractures and delayed fracture healing [42] and may therefore be vulnerable to injury from regular compressive loading through the cervical spine.

Frequent loading beyond capacity for adaptation or repair may also lead to early degenerative changes in bone and soft tissues [12]. A threshold of 250 Newtons of sustained cyclic loading (15% of failure stress, approximately 6MPa) applied to articular cartilage in vitro has been reported as a threshold above which cell death occurs and increases in proportion to the applied load [59]. Cell death in mature cartilage can lead to degradation of the tissue and is associated with onset of osteoarthritis [59]. Although the actual forces sustained by the cervical spine during water carrying have not been directly quantified, this study indicates that they are likely to exceed 250 Newtons for many individuals, when the weight of the head and effects of muscle contraction are added to the weight of water carried. Whilst pain, stiffness and functional impairment are clinical features of osteoarthritis, the correlation between symptoms such as pain and radio-graphically observed degenerative changes is not clearly established. Therefore future research should investigate the relationships between loading intensity, frequency and duration, history of physical loading exposure and symptoms such as neck or back pain and functional disability, rather than radiographic examination findings alone.

Guidance on good manual handling techniques for safety when pushing loads in high income countries suggest that worn wheelbarrow tyres and lack of grip padding on various types of equipment can increase the work of pushing and affect grip force and comfort [60,61]. This can be particularly plausible on sandy pathways such as those along which water was frequently carried by the participants in this study. Water is also an inherently unwieldy load, which moves within the containers during handling. Although the participants had clearly developed skills to lift and balance containers, maintenance of a secure grip would be difficult during sudden or unexpected posture changes, as might occur when walking along routes shared with vehicles and domestic animals.

Sudden or unexpected posture changes may lead to injury through generation of high peak compressive forces. These can occur due to muscle action on the spine [11] which in the cervical region is required to support the weight of the head and loads applied to it to prevent spinal buckling [62,63]. Rapidly or awkwardly lifting objects or accidents during manual handling can generate peak compressive forces higher than injury threshold, but may also create torsional, shear or bending moments which injure the spine if it is inadequately stabilised [11]. Hazards for slips, trips and falls include wet and uneven surfaces, obstacles, exposure to traffic, poor equipment and unwieldy loads [64,65], all of which were typical environmental and task related factors of domestic water carrying.

Distance walked between water source points and the home may be a useful indicator of exposure time to sustained compressive loading. As the distance walked by those who reported spinal pain was significantly less than those who did not, our results might indicate pain-related disability. People with spinal pain may experience difficulty carrying water over distance and be more likely to enlist the help of other family members and children, or continue to carry water only if it is accessible close to home. Such functional disability may have further implications for families, for example, by leading to a reduction in the usual volumes of water collected for household use to support health and adequate hygiene.

Our study suggests that the volume of water carried and environmental factors, particularly the incline or gradient of the path along which water is carried are likely to influence the physical work of water carrying as indicated by RPE. It also suggests that people reporting neck or head pain may be those who carry heavier containers and also perceive the task to be more difficult, as for head-loaders reporting head or neck pain, the differences in weight of water carried and RPE were almost statistically significant. Distance walked whilst carrying water, volume of water carried and path gradient are therefore important quantifiable factors which might be useful to indicate the physical work load of water carrying.

## Conclusions

This study has highlighted the potential association between spinal pain and water carrying in South Africa. This association is complex with water carrying probably contributing to the aetiology of spinal pain and spinal pain interfering with people's ability to carry water with potential impact on household water availability. Typical methods of carrying water containers as observed in this study impose physical loading with potential to produce symptoms typical of musculoskeletal disorders and related disability. Risk of musculoskeletal injury or pain may be high as it is usually a task for women and children, including those who may be affected by chronic poor health, and is often performed with inadequate equipment in potentially hazardous environments. Water carrying is not the only manual work performed by women and children in lower income countries and future research should also investigate the additional burden from other physical tasks.

Carrying distance could be used together with total volume or weight of water carried and path gradient to indicate the level of physical work imposed by water carrying. These factors together with the modified Borg scale and water carrying method should be investigated in future research, to better understand the type and strength of association between water carrying and health, particularly symptoms typical of musculoskeletal disorders such as pain and related disability. Identifying risk factors for musculoskeletal disorders and pain related to water carrying may also highlight appropriate interventions to reduce risk exposure.

Despite the small study size and associated lack of power, our preliminary findings still highlight the potential impact that carrying water may have on health, in particular through the effects of symptoms typical of musculoskeletal disorders, such as neck or back pain, and related functional disability. This is an important but neglected public health issue. There is a need for more research on the impact of water carrying on neck and back pain and how such pain impacts on the water carriers lives. There is also a need for research into how water can be carried in a way that reduces the potential for adverse im.pacts on musculoskeletal health.

## Abbreviations

RPE: Rating of Perceived Exertion; kg: kilograms; m: metre; RA: Research Assistant; GPS: Global Positioning System; SPSS 15.0: Statistical Package for the Social Sciences version 15.0; N: Newtons of force; m/s^2^: metre per second squared; CW/BW%: Container Weight to Body Weight percentage; HIV: Human Immunodeficiency Virus.

## Competing interests

The authors declare that they have no competing interests.

## Authors' contributions

JG conceived of the study and its design, collected, analysed and interpreted the data and drafted the manuscript. PH contributed to conception of the study, performed statistical analyses, participated in interpretation of the data and helped to draft the manuscript. PJ participated in the study conception, design and coordination, interpretation of data and helped draft the manuscript. All authors read, critically revised and approved the final manuscript.

## Supplementary Material

Additional file 1 **Criteria for identifying work phases**. Criteria used for identifying duration of work phases from observation of video material. Click here for file
