# Supplementary material for: Domestic water carrying and its implications for health: a review and mixed methods pilot study in Limpopo Province, South Africa
Source: Environ Health. 2010 Aug 26;9:52. doi: 10.1186/1476-069X-9-52 (PMC2939590; doi:10.1186/1476-069X-9-52)
Supplement: Additional file 1 — Criteria for identifying work phases. Criteria used for identifying duration of work phases from observation of video material. [file 1476-069X-9-52-S1.DOC]

Criteria for identifying work phases

| **Filling**  **Start:** Participant at water source, when participant first grasps (first) container with one or both hands to position it for cleaning **and/or** filling  ***OR*** point at which video capture of this subtask begins if it has already commenced  **Finish:** Participant initiates movement to get into a position to grasp and lift (first) container **or** returns to upright relaxed position prior to positioning for lift  ***OR*** last point of video-capture for this subtask |
| --- |
| **Lifting**  **Start:** point at which participant is first observed to initiate movement to begin getting into a position to grasp and lift (first) container  OR point at which video capture of this subtask begins if it has already commenced  **Finish:** participant has placed (final) container in position for carry ready for commencement of carry and resumed upright position  ***OR*** last point of video-capture for this sub task |
| **Carry**  **Start:** participant has completed lift, containers are in position for carry (for wheel barrow participant has grasped handles and initiates movement to lift wheelbarrow)  ***OR*** point at which video capture of this subtask begins if it has already commenced  **Finish:** participant arrives at destination, stops walking at point for container(s) to be lowered **(and** has grasped container to initiate lift and lowering of container if carrying on head)  For wheelbarrow, has lowered wheelbarrow in final position and resumed upright position  ***OR*** last point of video-capture for this subtask |
| **Lowering and placement**  **Start:** participant has stopped walking **and** initiates/has initiated lift of container for lowering if head carrying or is in position and begins movement toward grasping container to initiate lift for wheelbarrow  ***OR*** point at which video capture of this subtask begins if it has already commenced  **Finish:** participant has placed (final) container in final position (may include holding container while emptying) and resumed upright or relaxed position  ***OR*** last point of video-capture for this subtask |
| **Criteria for calculating sub-task time**  **25 frames per second:**  **Up to frame 12 = previous second**  **Frame 13 forward = subsequent session** |
